# The endocytic pathway for absorption of exogenous RNAs in *Verticillium dahliae*

**DOI:** 10.1002/mlf2.12149

**Published:** 2025-02-07

**Authors:** Chuanhui Liu, Chen Cui, Guanyin Zhou, Feng Gao, Jianhua Zhao, Huishan Guo, Yun Jin

**Affiliations:** ^1^ State Key Laboratory of Plant Genomics, Institute of Microbiology Chinese Academy of Sciences Beijing China; ^2^ CAS Center for Excellence in Biotic Interactions University of Chinese Academy of Sciences Beijing China; ^3^ Zhongmian Seed Technologies Co., Ltd Zhengzhou China

**Keywords:** endocytic pathway, pathogenic fungi, RNA uptake, small RNA

## Abstract

RNAi technologies have been exploited to control viruses, pests, oomycetes, and fungal phytopathogens that cause disasters in host plants, including many agronomically significant crops. Double‐stranded RNA (dsRNA) or small interfering RNA (siRNA) has been applied as a trigger for trans‐kingdom RNAi between hosts and fungi. However, it is unclear what process mediates RNA uptake by fungi. In this study, by using live‐cell imaging, we determined that exogenously synthesized RNA or small RNA (sRNA) was indiscriminately absorbed into *Verticillium dahliae*, a notorious pathogenic fungus. Moreover, the application of endocytic inhibitors or deletion of endocytic‐related genes reduced RNA uptake efficiency, showing that RNA absorption by fungal cells occurs mainly through endocytosis. In addition, we found that the endocytosed fluorescence‐labeled RNAs were partly colocalized with endosome marker genes. Overall, our research concluded that exogenous RNA could be assimilated by *V. dahliae* through the endocytic pathway. Unraveling this cytological mechanism underlying trans‐kingdom RNAi holds significant importance, especially considering the fact that RNAi‐based strategies targeting pathogenic fungi are increasingly prevalent in the realm of crop protection.

## Impact statement

The cytological mechanisms underlying trans‐kingdom RNAi remain largely elusive. In this study, we clearly showed that the uptake of RNAs by *Verticillium dahliae* occurs through endocytosis mediated by actin‐related genes and that endocytosed RNAs are destined in endosomes. This image‐based analysis provides visual evidence for the application of RNAi‐based methods for crop protection against pathogenic fungi.

## INTRODUCTION

Small RNAs (sRNAs) not only play roles in their own cells but also function as signal molecules to induce trans‐kingdom gene silencing between different species, such as plants and fungi. The trans‐kingdom sRNA trafficking between plants and interacting fungi is bidirectional, occurring not only from pathogenic fungi to host plants^1^, ^2^, ^3^, ^4^ but also from plants to fungi^5^. Subsequently, trans‐kingdom sRNAs have been investigated in other interaction systems, including host plants and rhizobia^6^, and the parasitic plant *Cuscuta campestris* and host plants^7^. Based on this naturally occurring trans‐kingdom communication between plants and phytopathogens, host‐induced gene silencing (HIGS) by the expression of double‐stranded RNA (dsRNA) in host plants to target the virulence genes of pathogens has been robustly established to defend against invading microbes, although this strategy has been applied earlier in disease control than the discovery of natural trans‐kingdom RNAi^8^. Moreover, exogenously synthesized dsRNA/sRNA, known as spraying‐induced gene silencing (SIGS), has been also applied to protect some host plants^9^, suggesting that this nontransgenic strategy is valuable as a supplement to HIGS. Recently, microbe‐induced gene silencing (MIGS) was developed by using a beneficial rhizospheric fungus, *Trichoderma harzianum*, to exploit RNAi in two soil‐borne pathogenic fungi, *Verticillium dahliae* and *Fusarium oxysporum* ^10^. This MIGS‐based biofungicide is promising for crop protection against phytopathogens due to its sustainability and relatively low cost.

In the process of trans‐kingdom RNAi, a critical question that needs to be addressed is how these sRNAs are trafficked between species. There is evidence that both prokaryotic and eukaryotic microbes transport RNA via extracellular vesicles (EVs), such as exosomes and microvesicles. It has been shown that microbial EVs are enriched with sRNAs and might be universal mediators of communication between different organisms^11^. However, others have shown RNAs, which are abundant in the extracellular space, mediated communication^12^, ^13^. Among these nonvesicular forms of RNAs, microRNAs (miRNAs) are usually associated with the Argonaute (AGO) protein^13^, ^14^, ^15^. In addition, miRNAs can be transported in plasma and delivered to recipient cells by high‐density lipoproteins^16^. That is, trans‐kingdom RNAs are packaged and protected from degradation by EVs or protein particles.

Another question is how these trans‐kingdom RNAs are taken up by recipient cells. In animal cells, these RNAs enclosed in EVs are internalized through surface binding or other uptake pathways, including micropinocytosis, phagocytosis, caveolae, clathrin, lipid raft, or membrane fusion pathways^17^, ^18^. In addition to EVs carrying RNAs, other forms of exogenous RNAs are internalized by endocytosis in some species. For instance, in the red flour beetle, the cellular uptake of dsRNA relies on clathrin‐dependent endocytosis^19^. In *Drosophila* S2 cells, dsRNA is internalized by scavenger receptor‐mediated endocytosis^20^, ^21^. In plants, both clathrin‐mediated endocytosis (CME) and clathrin‐independent endocytosis (CIE) have been reported^22^. However, only recently has CME been reported to function in fungal EV transport of sRNAs into plant cells^23^. In addition to animal and plant cells, endocytosis has also been investigated in yeast^24^, ^25^ and some filamentous fungi in which endocytosis and exocytosis play roles in apical growth^26^, ^27^, ^28^. Only in the white mold phytopathogen *Sclerotinia sclerotiorum* is CME reportedly involved in the uptake of exogenous dsRNA^29^. However, what is the role of endocytosis in other fungi and how the plant EV‐containing sRNAs enter fungal cells are largely unknown.

We have previously shown that sRNAs from plants or rhizospheric beneficial fungi can enter *V. dahliae* hyphae and downregulate the expression of virulence genes, thus reducing their pathogenicity^5^, ^10^, ^30^. In this study, we examined how exogenous RNAs enter fungal cells. We identified endocytosis‐related actin‐mediated uptake of exogenous long and small RNAs, which can partly colocalize with endocytic vesicles. We provided image‐based cytological evidence that exogenous RNAs can enter fungal cells through the endocytosis pathway in *V. dahliae*.

## RESULTS

### Exogenously synthesized RNAs indiscriminately enter fungal hyphae

**Figure 1:**
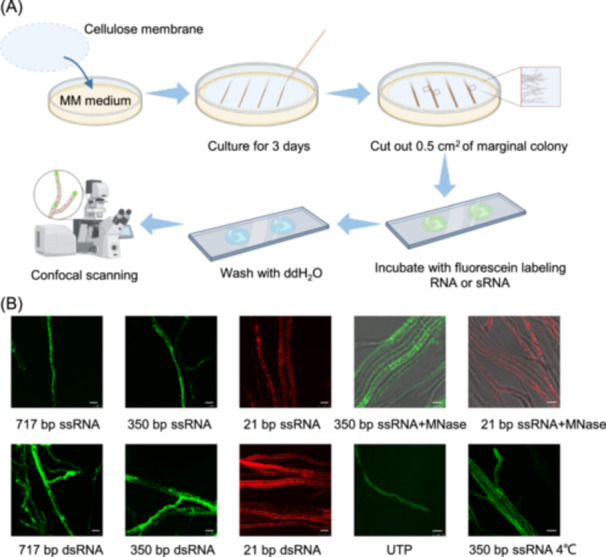
Exogenously synthesized RNAs can be absorbed by *Verticillium dahliae* hyphae. (A) An experimental flow chart to display the uptake assay. Created with BioRender.com (2024). Retrieved from https://app.biorender.com. (B) Fluorescence microscopy observation of *V. dahliae* hyphae after coincubation with synthesized RNA or sRNA. Green fluorescence represents fluorescein‐labeled RNAs, and red fluorescence represents Cy3‐labeled sRNAs. Bar = 10 μm. UTP was used as a negative control. MM medium, minimal medium; MNase, micrococcal nuclease; sRNA, small RNA; dsRNA, double‐stranded RNA; ssRNA; single strand RNA.

To determine the process by which exogenous RNAs are transmitted into fungal cells, we first established a visible experimental system by using in vitro synthetized RNA with fluorescein‐labeled UTP. A 350 bp fragment of the *VdH1* gene or a 717 bp fragment of the *RFP* gene was cloned and inserted into the pGM‐T vector with the T7 promoter in both the sense (+) and antisense (−) orientations. In vitro transcription obtained single strand (ss) VdH1 (+), ssVdH1 (−), ssRFP (+), and ssRFP (−). dsRNAs (dsVdH1 and dsRFP) were obtained by annealing (+) and (−) strands at 72°C for 10 min. The labeled ssRNAs and dsRNAs were then coincubated with wild‐type V592 hyphae for 30 min before observation under a confocal microscope. Fluorescein‐labeled UTP was used as a control. An experimental flow chart is shown in Figure 1A to better display the uptake assay we conducted. Compared with the weak and diffuse fluorescence in the control (Figure 1B), strong dot‐gathered fluorescence was observed in the ss‐ or ds‐ RNA‐incubated V592 hyphae (Figure 1B). To assess whether RNAs are internalized via an active or diffuse process, the temperature dependence of RNA uptake was examined. As shown in Figure 1B, no dot‐gathered fluorescence was observed in the interior of hyphae incubated with ssRNAs (ssVdH1) at 4°C, indicating that the uptake of exogenous RNAs by *V. dahliae* is not a passive diffusion process. We further treated hyphae with MNase and found that dot‐gathered fluorescence was still observable, excluding the influence of external unabsorbed RNAs (Figure 1B). In addition to the long RNA strand, exogenously synthesized 21‐bp ssRNAs (Cy3‐labeled miR166) and 21‐bp dsRNAs (Cy3‐labeled siVdH1) were also able to enter fungal cells (Figure 1B). After MNase treatment, dot‐gathered fluorescence was still observed in the interior of hyphae for 21‐bp dsRNAs incubation (Figure 1B). These findings indicate that both exogenously synthesized long and small RNAs, regardless of whether they are double‐ or single‐stranded, can enter *V. dahliae* hyphal cells.

### Cellular uptake of exogenously synthesized RNAs is reduced upon treatment with endocytosis inhibitors

**Figure 2:**
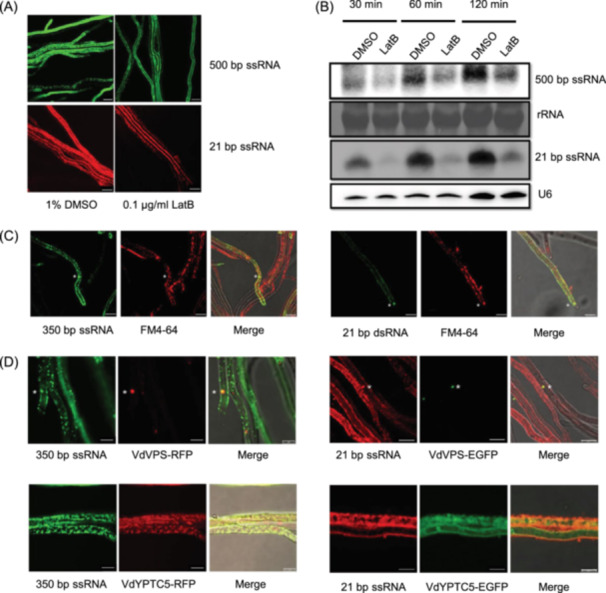
Exogenous RNA uptake by fungal hyphae occurs primarily via the endocytosis pathway. (A) Observation of *V. dahliae* hyphae treated with LatB after coincubation with synthesized RNA or sRNA. Green fluorescence represents fluorescein‐labeled RNAs, and red fluorescence represents Cy3‐labeled sRNAs. Bar = 10 μm. (B) Northern blot analysis of exogenous RNAs absorbed by fungal protoplasts under LatB treatment. Dimethyl sulfoxide (DMSO) was used as a negative control. (C) Synthetic RNAs partly colocalized with FM4‐64‐designated endocytic vesicles. Green fluorescence represents fluorescein‐labeled RNA and sRNA. Red fluorescence represents FM4‐64‐labeled membrane structures. The asterisk indicates the colocated endocytic vesicles. Bar = 7.5 μm. (D) Synthetic long or small ssRNAs partly colocalized with endosome marker genes. For long ssRNAs, green fluorescence represents fluorescein‐labeled RNAs, and red fluorescence represents VdVPS or VdYPTC5 protein localization. Bar = 5 μm. For small ssRNAs, green fluorescence represents VdVPS or VdYPTC5 protein localization, and red fluorescence represents fluorescein‐labeled sRNAs. Bar = 7.5 μm. The asterisk indicates the colocated early endosome.

To determine whether the uptake of exogenous RNAs is dependent on endocytosis, we treated hyphae with latrunculin B (LatB), an inhibitor of endocytosis. After treatment with 0.1 µg/ml LatB for 30 min, the hyphae were incubated with 500‐bp fluorescein‐labeled ssRNAs (ssRFP) and 21‐bp ssRNAs (Cy3‐miR166). Compared with the control hyphae treated with 1% DMSO, the LatB‐treated hyphae showed reduced dotted fluorescence, suggesting decreased RNA uptake (Figure 2A). For quantitative detection of RNA uptake, we prepared protoplasts of V592 to obtain consistent fungal cells. Protoplasts of V592 were pretreatment with LatB or DMSO for 30 min before being incubated with 500‐bp ssRNAs (ssRFP) or 21‐bp ssRNAs (Cy3‐miR166) for 30, 60, or 120 min. Northern blot analysis showed lower levels of RNAs in protoplasts with LatB treatment (Figure 2B), further demonstrating that the endocytosis inhibitor LatB impaired the uptake of exogenous RNAs by *V. dahliae*.

### Exogenous RNA is partly colocalized with endosomes after internalization in fungal cells

To further investigate the distribution of these RNAs taken up by recipient cells, FM4‐64 was used to indicate the plasma structure, as it can enter cells through binding to the bilayer of the plasma membrane^31^. The fluorescein‐labeled 350‐bp ssRNAs (ssVdH1) were incubated with V592 hyphae for 30 min and then dyed with FM4‐64. Obviously, overlapping fluorescence was observed at the plasma membrane (Figure 2C). Similarly, we detected overlapping fluorescence when the 21‐bp dsRNAs, generated by digestion of the fluorescein‐labeled 717‐bp dsRFP with ShortCut RNase III, were incubated with V592 hyphae for 30 min and then dyed with FM4‐64 (Figure 2C). Notably, overlapping dot fluorescence was observed in the cytoplasm, indicating that the RNAs colocalized with the FM4‐64‐designated endocytic vesicles (Figure 2C, asterisks). These results demonstrate that exogenous RNAs are likely enclosed in endocytic vesicles after they are endocytosed to fungal cells.

Endosomes are plasma‐coated vesicular structures, including early endosomes (EEs) and late endosomes (LEs)^22^, which bind the small GTPases Rab5 and Rab7^32^, ^33^, ^34^, respectively, to participate in the endocytic pathway. In filamentous fungi, receptor‐mediated endocytosis also results in the processing of cargos in EEs and LEs^27^. To determine whether exogenous RNA is localized to EEs or LEs after being transmitted to hyphae, we cloned *VdVPS* and *VdYPTC5*, which encode the small GTPases Rab5 and Rab7, respectively. *VdVPS* and *VdYPTC5* were fused with EGFP or RFP to indicate the localization of EEs and LEs, respectively. As shown in Figure 2D, compared to a few VdVPS‐RFP‐labeled EEs observed, a large amount of VdYPTC5‐RFP‐labeled LEs was distributed in the hyphae (Figure 2D). Accordingly, coincubation of the 350‐bp ssRNAs (ssVdH1) with VdVPS‐RFP or VdYPTC5‐RFP showed that the 350‐bp ssRNAs were most colocalized with small GTPases in LEs, while they could be also in EEs (Figure 2D). Similarly, the 21‐bp Cy3‐miR166 was also colocalized with VdVPS‐EGFP‐labeled EEs, but most with VdYPTC5‐EGFP‐labeled LEs (Figure 2D). These results demonstrate that the endocytic uptake of extracellular RNAs is transmitted into EEs, and particularly, LEs in fungal cells.

### VdCapA and VdEND3 are involved in endocytosis

As actin proteins are vital components of the cytoskeleton that are involved in endocytosis and vesicle trafficking processes in fungi^35^, ^36^, ^37^, we searched for endocytosis‐related actin genes in *V. dahliae* and found two genes, *VdCapA* and *VdEND3*, encoding F‐actin‐capping protein subunit alpha and actin cytoskeleton‐regulatory complex proteins, respectively (Figure S1A). We knocked out the two genes and generated the deletion mutants VdΔ*capa* and VdΔ*end3*, as well as the double mutant VdΔ*capaend3*, which were all confirmed by Southern blotting (Figure S1B).

**Figure 3:**
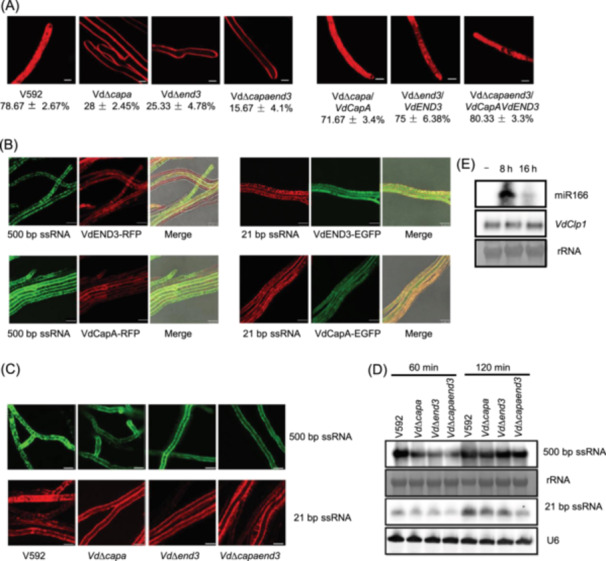
*VdCapA* and *VdEND3* are two endocytosis‐related genes involved in exogenous RNA uptake. (A) The endocytosis rate decreased in endocytic knockout mutants Vd∆*capa*, Vd∆*end3*, and Vd∆*capaend3*. The numbers represent the percentages of mycelia with obvious fluorescence, and the standard deviation was calculated based on the results of three repeated experiments. Each group contained 100 mycelia. Bar = 2.5 μm. Red fluorescence represents FM4‐64‐labeled membrane structures. (B) Synthetic long ssRNAs or sRNAs partly colocalized with VdCapA and VdEND3. For long ssRNAs, green fluorescence represents fluorescein‐labeled RNAs, and red fluorescence represents VdCapA or VdEND3 protein localization. For small ssRNAs, green fluorescence represents VdCapA or VdEND3 protein localization, and red fluorescence represents fluorescein‐labeled RNAs. Bar = 7.5 μm. (C) The absorption of exogenous RNAs reduced in Vd∆*capa*, Vd∆*end3*, and Vd∆*capaend3*. Green fluorescence represents fluorescein‐labeled RNAs, and red fluorescence represents Cy3‐labeled sRNAs. Bar = 2.5 μm. (D) Northern blot analysis of exogenous RNAs absorbed by fungal protoplasts in the wild‐type and mutant strains Vd∆*capa*, Vd∆*end3*, and Vd∆*capaend3*. (E) Northern blot analysis of exogenously synthesized miR166 absorbed by protoplasts of V592 and the expression of target gene *VdClp1*.

As FM4‐64 is an endocytic membrane‐selective marker, we treated hyphae with FM4‐64 for 1 min to characterize the role of *VdCapA* and *VdEND3* in endocytosis. As shown in Figure 3A, the plasma membrane and endosomes of most hyphae showed fluorescence signals in the wild‐type V592 but not in the VdΔ*capa*, VdΔ*end3*, or VdΔ*capaend3* deletion mutants (Figure 3A), suggesting that the internalization of FM4‐64 was impaired in the mutants. Among randomly selected 100 hyphae, approximately 78.67% of the V592 hyphae showed obvious fluorescence, whereas more than 70% of the VdΔ*capa* and VdΔ*end3* mutants showed nearly no fluorescence inside the hyphae. Specifically, only about 28% and 25.33% of the hyphae showed fluorescence in VdΔ*capa* and VdΔ*end3*. The proportion of fluorescence hyphae reduced to 15.67% in the VdΔ*capaend3* double mutant (Figure 3A). The results indicate that the efficiency of endocytosis in VdΔ*capa*, VdΔ*end3*, and VdΔ*capaend3* was significantly reduced. All the complementation lines rescued the uptake defects and the fluorescence was almost the same as that in V592 (Figure 3A). These results demonstrate that the endocytosis‐related actin genes, *VdCapA* and *VdEND3*, play a role in endocytosis that mediates the internalization of FM4‐64 into fungal cells.

### RNA uptake is attenuated in endocytic mutants

In order to determine whether *VdCapA* and *VdEND3* contribute to RNA uptake, we first cloned the coding sequences (CDSs) of *VdCapA* and *VdEND3* and constructed VdCapA‐EGFP/RFP and VdEND3‐EGFP/RFP for fungal transformation. Protein localization of VdCapA‐EGFP/RFP and VdEND3‐EGFP/RFP was observed under confocal microscopy. The images showed that both VdCapA and VdEND3 were located in fungal hyphae with dot‐gathered distribution (Figure 3B). The hyphae of VdCapA‐EGFP/RFP and VdEND3‐EGFP/RFP were coincubated with 500‐bp ssRNAs (ssRFP) or 21‐bp ssRNAs (Cy3‐miR166), and confocal microscopy images showed that partial colocalization of the exogenous RNAs with VdCapA and VdEND3 (Figure 3B), implicating that the two endocytosis‐related actin genes might take part in uptake of RNAs.

We thus assessed the efficiency of RNA uptake in the wild‐type and the knockout mutant strains. The fluorescein‐labeled 500‐bp ssRNAs were used for incubation. While the dotted green fluorescence in V592 hyphae was clearly visible, it was reduced in the VdΔ*capa*, VdΔ*end3*, and more obviously, in the VdΔ*capaend3* double mutant (Figure 3C). To quantitatively detect the RNA uptake efficiency, protoplasts of V592 and the knockout mutants were prepared for incubation with equal amounts of 500‐bp ssRNAs for 60 or 120 min, followed by MNase treatment. Northern blot analysis showed reduced RNA uptake in the three mutant strains compared with wild‐type V592 at the 60 min time point (Figure 3D). However, after 120 min of incubation, the RNA hybridization signals were indistinguishable between V592 and the mutants, even in the double mutant (Figure 3D), suggesting that the existence of other endocytosis processes, for example, CME, assumed the uptake of long RNA in the absence of VdCapA and VdEND3.

We next tested with the 21‐bp Cy3‐miR166 ssRNAs. While the dotted Cy3‐miR166 red fluorescence in V592 hyphae was clearly visible, it was greatly reduced in the VdΔ*capa*, VdΔ*end3*, and especially, in the VdΔ*capaend3* double mutant (Figure 3C). To quantitatively detect the RNA uptake efficiency, protoplasts of V592 and the knockout mutants were prepared for incubation with equal amounts of Cy3‐miR166 RNAs for 60 or 120 min, followed by MNase treatment. Northern blot analysis revealed that the uptake of the 21‐bp ssRNAs increased along with incubation time. However, compared to wild‐type V592, the RNA uptake was reduced in VdΔ*capa*, VdΔ*end3*, and especially, VdΔ*capaend3* mutant in both time points (Figure 3D), consistent with the fluorescent images (Figure 3C), indicating their main but redundant roles in uptake of sRNAs. Taken together, all these results reveal that VdCapA and VdEND3 mainly responsible for both long and small RNA endocytosis, and the uptake of RNAs presumably involves the hierarchical action of multiple endocytosis processes in *V. dahliae*.

We have previously found that plant‐exported miR166 mediated silencing of *VdClp1* gene in fungal cells during *V. dahliae* infection^38^. We then examined whether uptake of exogenous miR166 could degrade *VdClp1* in wild‐type V592. Protoplasts of V592 were incubated with synthetic miR166 for 8 and 16 h, followed by MNase treatment. Northern blot analysis detected a large amount of miR166 in V592 protoplasts collected at 8 h, but greatly reduced for 16 h incubation (Figure 3E). However, similar accumulation levels of *VdClp1* mRNA were detected at either time point (Figure 3E), demonstrating that the synthesized miR166 failed to silence *VdClp1*. This result suggests that exogenously synthesized RNAs were unable to induce silencing of the fungal target gene, presumably due to the lack of modification as their natural synthesis in plant cells. This phenomenon requires further investigation.

### The uptake of exogenous RNA in *V. dahliae* is also attenuated upon a CME inhibitor treatment

**Figure 4:**
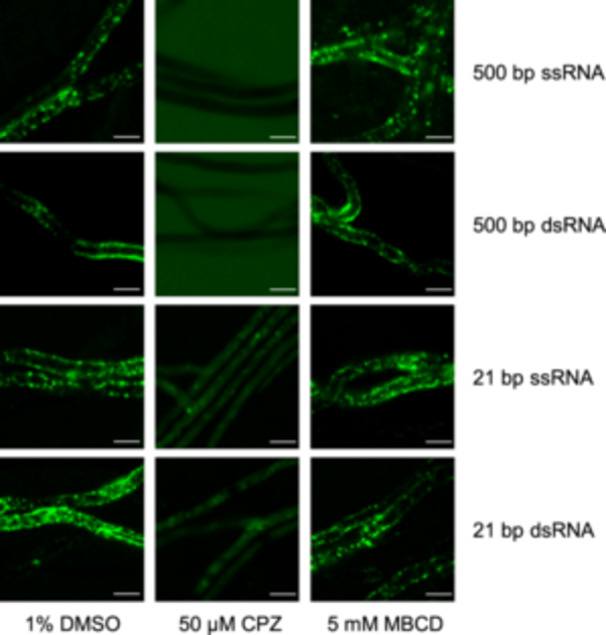
Clathrin‐mediated endocytosis inhibitor chlorpromazine (CPZ) inhibits exogenous RNA uptake in *V. dahliae*. Observation of *V. dahliae* hyphae treated with 50 μΜ CPZ or 5 mM methyl‐beta‐cyclodextrin (MBCD) before coincubation with synthesized RNA or sRNA. 1% DMSO was used as negative control. Green fluorescence represents fluorescein‐labeled RNAs. Bar = 5 μm.

In addition to the actin‐related endocytosis in cellular internal distribution of the RNAs, we further explored whether CME was involved in RNA uptake in *V. dahliae*, as CME has been found to be the dsRNA uptake mode in some phytopathogens^29^. We treated hyphae with chlorpromazine (CPZ) or methyl‐beta‐cyclodextrin (MBCD), which inhibits clathrin‐dependent or clathrin‐independent processes, respectively. After treatment with CPZ or MBCD for 60 min, the hyphae were incubated with 500‐bp (ssRFP or dsRFP) or 21‐bp (miR166 or ds‐siVdH1) fluorescein‐labeled ssRNAs or dsRNAs for 60 min. Compared with the control hyphae treated with 1% DMSO, the CPZ‐treated hyphae showed significantly reduced dotted fluorescence, almost invisible when incubated with either 500‐bp or 21‐bp RNAs, suggesting that the RNA uptake was severely impaired (Figure 4). Whereas, the distribution of the fluorescence in MBCD‐treated hyphae did not appear to differ greatly from that in DMSO‐treated hyphae (Figure 4). The inhibition of uptaken exogenous RNAs by the CME inhibitor demonstrates that CME is the main type of RNA uptake in *V. dahliae*. Unfortunately, neither clathrin light nor heavy chain genes were successfully knocked out in *V. dahliae* in our study, restricting further validation and identification.

### VdCapA and VdEND3 are required for fungal pathogenicity

Finally, we investigated the functions of *VdCapA* and *VdEND3* in fungal growth and pathogenicity. Compared to wild‐type V592, the growth rates were reduced for VdΔ*capa* and VdΔ*end3*, and more remarkable for the double mutant VdΔ*capaend3* (Figure S2A). Melanin microsclerotia formation was affected in VdΔ*end3* and VdΔ*capaend3* (Figure S2A), showing the involvement of *VdEnd3* in microsclerotium development. To investigate the roles of *VdCapA* and *VdEND3* during the colonization of *V. dahliae*, the penetration abilities of VdΔ*capa*, VdΔ*end3*, and VdΔ*capaend3* were examined by incubating the fungal strains on a cellophane membrane laid on minimal media. At 3 dpi, fungal growth was observed (Figure S2B) and the cellophane membrane was removed, followed by another 3 days of culture. Hyphal penetration from the cellophane membrane and growth on media were detected for wild‐type V592 and the mutant strains (Figure S2B), indicating that *VdCapA* and *VdEND3* are dispensable for penetration peg formation for initiation of infection in *V. dahliae*. Plant infection assay showed a great reduction in wilt disease symptoms (Figure S2) in cotton plants inoculated with either mutant, especially the double mutant strains, compared with those inoculated with V592 and the complemented strains (Figure S2). These findings are in agreement with the previous reports for other pathogenic fungi, such as *Magnaporthe oryzae* ^35^, ^36^ and *Ustilago maydis* ^39^, which show endocytosis is essential for pathogenicity in *V. dahliae*.

## DISCUSSION

**Figure 5:**
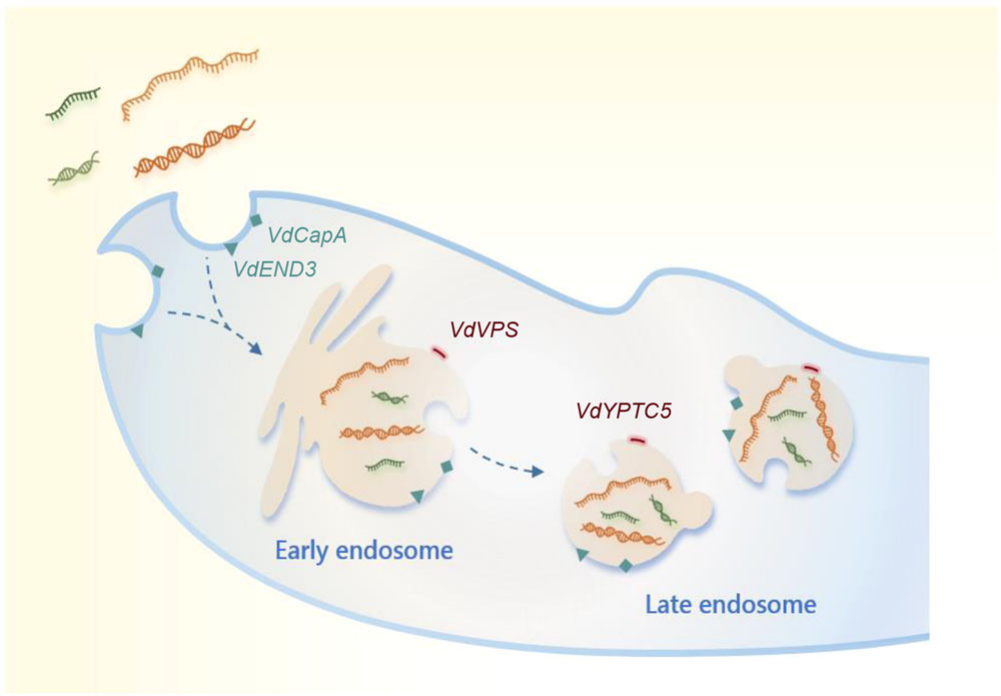
Model of the endocytic pathway for exogenous RNAs absorption in *V. dahliae*. Uptake of exogenous long and small RNAs is mediated by actin‐related endocytosis genes *VdCapA* and *VdEND3*, and the endocytosed RNAs are processed to early endosomes and late endosomes marked by *VdVPS* and *VdYPTC5*.

Here, we report the identification of endocytosis‐related actin‐mediated uptake of exogenous long and small RNAs, which can partly colocalize with endocytic vesicles, as well as EEs and LEs that participate in the endocytic pathway (Figure 5). Endocytosis participates in cellular signal transduction. Signal molecules, such as proteins or RNAs, are trafficked and recycled through endocytosis to participate in cell‐to‐cell communication^40^. By using an in vitro coincubation assay, we first proved that the uptake of RNA by *V. dahliae* hyphae was nonselective. Long or small RNAs, dsRNA, or ssRNA could enter *V. dahliae* hyphal cells (Figure 1B). Upon treatment with an endocytosis inhibitor LatB and CME inhibitor CPZ, we found that the cellular internalization of exogenously synthesized RNAs was reduced, and the CME‐dependent uptake process played a major role in RNA uptake (Figures 2A,B and 4). In addition, by confocal microscopy observation of FM4‐64, which is used as a tool for analyzing endocytosis in living fungal hyphae, we located the absorbed exogenous RNA in FM4‐64‐designated vesicles (Figure 2C). Moreover, we found that two actin proteins, VdCapA and VdEND3, were required for endocytosis. After incubation with FM4‐64 or RNA, single and double deletions of *VdCapA* and *VdEND3* significantly reduced the uptake efficiency (Figure 3A,C,D).

During the process of endocytosis, RNA was partly colocalized with VdCapA and VdEND3 (Figure 3B). Moreover, we used fluorescent‐tagged RAB plasmids to specifically identify the compartment and found that the RNA‐packaged vesicles were partly colocalized with the endosome marker genes *VdVPS* and *VdYPTC5*, which are homologous to Rab5 and Rab7 (Figure 2D), indicating the subcellular location of the endocytosed RNA. Thus, the following questions remain: can all the RNAs in early and late endosomes be released into the cytosol? Up to now, almost all studies show that only a very small amount of cargo is released into the cytosol. In contrast, a large part of the cargo is recycled out of the cell or degraded in lysosomes^41^, ^42^, ^43^. Whether the amount of endocytosed sRNA is sufficient to trigger RNAi needs to be further investigated.

HIGS and MIGS are known as trans‐kingdom and interspecies RNAi‐based strategies for crop protection, regardless of the presence of RNA molecules from the host or other microbes. These strategies act well in host protection, preventing severe symptoms caused by pathogenic microbe attack^44^, ^45^. Notably, in the natural infection process, the population and abundance of plant‐derived miRNAs in hyphae isolated from infected cotton are different from those of endogenous cotton miRNAs^5^, ^46^. This finding suggests that there is a mechanism underlying sRNA sorting, either through selective secretion from plants or release from endosome vesicles in fungi, rather than through the uptake of naked RNAs. SIGS has also been reported to have a protective effect on some host plants. Whether the uptake of exogenously synthesized RNAs truly induces silencing of target genes needs to be cautiously verified, as uptake of synthesized miR166 did not induce target gene silencing, despite the high accumulation level of miR166 detected (Figure 3E). Nevertheless, in addition to sRNAs/dsRNAs, which have the potential to trigger RNAi in recipient cells, long RNAs (mRNAs or long noncoding RNAs) that are trafficked between interacting organisms have recently been shown to have robust trans‐kingdom functions^47^. More importantly, RNA therapy or mRNA vaccines based on RNA delivery have been successfully applied^43^. Our finding of endocytosis‐mediated synthesized RNA uptake and trafficking in fungi provides useful evidence for RNA application in plant‐fungi interaction systems and are not limited to trans‐kingdom RNAi for crop plant protection.

## MATERIALS AND METHODS

### Fungal isolates, culture conditions, penetration, and infection assays

The virulent defoliating *V. dahliae* strain V592, isolated from cotton in Xinjiang, China, was used in this study. This isolate was stored as a microconidial suspension in 20% glycerol at −80°C and was reactivated by growing on potato dextrose agar (PDA) plates at 26°C before use^48^. The penetration assay^49^ and the infection assay^5^ were performed as previously described in our laboratory. For the penetration assay, a sterilized cellophane membrane was overlaid onto a minimal medium (MM). After 3 days of incubation of the cultures, the cellophane membrane was removed, followed by another 2 days for penetration detection. For the infection assay, disease progression was recorded after approximately 3 weeks of incubation. The experiments were repeated independently at least three times.

### Fluorescein‐labeled RNA incubation with fungal hyphae

The spores were inoculated on MM media supplemented with a cellulose membrane for 3 days. A marginal colony of approximately 0.5 cm^2^ was cut, and 50 μl of 5 μM RNA or sRNA was added to the fungal hyphae adhered to the cellulose membrane. The mixture was incubated at 26°C for 30 min. Then, the sections were rinsed with distilled water 3 times. The hyphae were incubated with 75 U of MNase for 30 min at 37°C in the dark to digest the RNA outside the fungal cells. The samples were rinsed with distilled water before observation under a confocal microscope.

### Cloning, constructs, and transformation

To obtain the constructs T7‐VdH1 and T7‐RFP, *VdH1* was amplified from V592 cDNA with the primers VdH1‐350‐F/R. *RFP* was amplified from the constructs with the primers RFP‐717‐F/R or RFP‐500‐F/R. The fragment was inserted into the pGM‐T vector.

To obtain pOliC‐VPS/YPTC5/VdCapA/VdEND3‐EGFP/RFP constructs with the *OliC* promoter, cDNA sequences were amplified and fused to *Pac*I/*Bam*HI‐linearized pOliC‐Nat‐OliC‐EGFP/RFP‐TrpC^50^. To generate pTef‐VdCapA/VdEND3‐Neo/Chl with the *Tef* promoter, the cDNA sequences were amplified and fused to *Bam*HI/*Eco*RI‐linearized pTef‐Neo/Chl‐TrpC. All of these constructs were generated by using ClonExpress II (Vazyme). The primers used are shown in Table S1.

To generate the pGKO‐HPT‐VdCapA and pGKO‐HPT‐VdEND3 knockout plasmids, upstream and downstream genomic sequences were amplified with the following primer pairs: VdCapA‐up‐F/R and VdCapA‐dn‐F/R; and VdEND3‐up‐F/R and VdEND3‐dn‐F/R (Table S1). The upstream and downstream sequences were inserted into two sides flanking the hygromycin‐resistant cassette of the pGKO vector with the USER enzyme. To generate the double knockout mutant, the vector pGKO‐Nat was reconstructed by replacing the hygromycin‐resistant cassette with a nourseothricin‐resistant cassette. Then the plasmid pGKO‐Nat‐VdEND3 was constructed as abovementioned, and transformation was performed as described previously^48^. Fungal transformation was performed as previously described^51^.

### Nucleic acid extraction and blotting

Total DNA and RNA were extracted as previously described in our laboratory^10^. For Southern blot analysis, 20–30 µg of DNA was separated by 0.9% agarose gel electrophoresis and transferred to a nylon membrane (Amersham Hybond‐N+ membrane) (RPN303B; GE). The probes were labeled by biotin‐11‐dUTP (R0081; Thermo Fisher) and denatured before being added to the hybridization solution (PerfectHyb Plus hybridization buffer) (H7033‐1 L; Sigma). Hybridization was performed overnight at 65°C. For sRNA detection, 30–40 µg of total RNA was separated on a 17% denaturing polyacrylamide gel and transferred to a nylon membrane. The 21‐nt probes with the 3′‐end labeled biotin were synthesized by RuiBiotech. Hybridization was performed overnight at 42°C. For mRNA detection, 10–20 µg of total RNA was separated on 1.2% agarose gel added with 1% formaldehyde and transferred to a nylon membrane. Denatured probes were added to the hybridization solution and hybridized overnight at 65°C. The hybridization signals were detected by using a chemiluminescent nucleic acid detection module kit (89880; Thermo Fisher). The images were obtained by using a fully automatic chemiluminescence image analysis system (Tanon‐4600SF; Tanon).

### In vitro RNA transcription

In vitro transcription of fluorescein‐labeled RNA was performed with Fluorescein RNA Labeling Mix (Roche) according to the manufacturer's instructions. T7‐VdH1 and T7‐RFP were used as templates to synthesize single‐strand ±VdH1/RFP RNA. dsRNA was produced by incubating equal amounts of ±stranded RNA at 72°C for 10 min, followed by gradual cooling to room temperature. Fluorescein‐labeled small RNA was produced with dsRNA cut by ShortCut Ribonuclease III (NEB) according to the manufacturer's instructions.

### Light microscopy

A confocal laser microscope (Leica TCS SP8; Leica Microsystems) with a ×100 oil immersion objective lens was used in this study for the fluorescence observation. For EGFP, the excitation wavelength is 488 nm, and the emission filters are 500 to 550 nm. For RFP and FM4‐64, the excitation wavelength is 561 nm, and the emission filters are 570 to 670 nm. Confocal images were captured with a Leica hybrid detector and analyzed with Leica LAS AF software.

### Staining of fungi and treatment with LatB

For plasma membrane staining, FM4‐64 (Thermo Fisher) was used according to the manufacturer's protocol. The spores were inoculated on MM media supplemented with a cellulose membrane for 3 days. A marginal colony of approximately 0.5 cm^2^ was cut, and 10 μg/ml FM4‐64 was added. After 5 min of incubation, the cells were rinsed three times with distilled water.

For LatB treatment, spores were inoculated on MM supplemented with cellulose membranes for 3 days. A marginal colony of approximately 0.5 cm^2^ was removed, and 50 μg/ml LatB (dissolved in DMSO) or 1% DMSO (negative control) was added. The cultures were incubated for 30 min, washed three times with distilled water and subjected to microscopic observation.

### Protoplast preparation and incubation with RNA

For the 10 ml lyase mixture, 20 µl of 5 U/μl zymolyase, 0.2 g of lysing enzyme, and 0.2 g of driselase were dissolved in water and infiltrated through a 0.45 μm filter. A total of 10^8^ spores were cultured in 100 ml of CM for 48 h and then infiltrated with Miracloth to collect the fungal hyphae. Hyphae (0.5 g) of *V. dahliae* were incubated with 10 ml of prepared lyase for 6–8 h on a shaker. The cells were checked every hour until the cell wall was thoroughly digested and round protoplasts formed. Then, the samples were infiltrated with 22–25 μm miracloth to collect the filtrate. The mixture was centrifuged at 700*g* for 5 min to collect the protoplasts. The mixture was rinsed three times with 1.2 M precooled KCl. The protoplast was resuspended to 10^8^/ml with 1.2 M KCl. Then, 50 μl of 5 μM RNA or sRNA was added to 1 ml of protoplast, which was shaken at 40 rpm for 30, 60, or 120 min. The mixture was centrifuged at 700*g* for 5 min to collect the protoplasts. The mixture was rinsed three times with 1.2 M precooled KCl. The protoplasts were resuspended in 500 μl of 1.2 M KCl, 75 U of MNase was added, and the mixture was incubated at 37°C and 40 rpm for 30 min. The mixture was centrifuged at 700*g* for 5 min to collect the protoplasts. The mixture was rinsed three times with 1.2 M precooled KCl.

### Treatment with chemical inhibitors CPZ and MBCD and fluorescein‐labeled RNA incubation

For chemical inhibitor treatment, spores were inoculated on MM supplemented with cellulose membranes for 3 days. A marginal colony of approximately 0.5 cm^2^ was removed, and 50 μM chlorpromazine (dissolved in DMSO) or 5 mM methyl‐beta‐cyclodextrin (dissolved in DMSO) was added; 1% DMSO was used as a negative control. The cultures were treated for 60 min of treatment before RNA incubation. Fluorescein‐labeled 500 bp dsRFP/ssRFP and 21 bp ds‐siVdH1 (AUCCCUCGAAACGCGUGAGCU)/ss‐miR166 (UCGGACCAGGCUUCAUUCCCC) were used. The other steps are the same as the “Fluorescein‐labeled RNA incubation with fungal hyphae” section.

## AUTHOR CONTRIBUTIONS

**Chuanhui Liu:** Conceptualization (equal); data curation (equal); formal analysis (equal); investigation (lead); validation (lead). **Chen Cui:** Conceptualization (equal); data curation (equal); formal analysis (equal); investigation (lead). **Guanyin Zhou:** Investigation (supporting); funding acquisition (supporting). **Feng Gao:** Investigation (supporting); funding acquisition (supporting). **Jianhua Zhao:** Formal analysis (supporting); investigation (equal); writing—original draft (supporting); writing—review and editing (supporting). **Huishan Guo:** Conceptualization (lead); formal analysis (equal); funding acquisition (lead); project administration (lead); supervision (lead); writing—review and editing (equal). **Yun Jin:** Conceptualization (equal); formal analysis (equal); funding acquisition (supporting); writing—original draft (lead); writing—review and editing (equal).

## ETHICS STATEMENT

No animals or humans were involved in this study.

## CONFLICT OF INTERESTS

The authors declare no conflict of interests.

## Supporting information


**Figure S1.** Identification of *V. dahliae* knockout mutants of endocytosis‐related genes. (A) Protein structures of VdCapA and VdEND3. Pink represents the low‐complexity region, purple represents the EH domain, and green represents the coiled‐coil domain. (B) Southern blot analysis of knockout mutants. The restriction enzymes *Bam*HI and *Eco*RI were used to digest genomic DNA.
**Figure S2.** Phenotype and pathogenicity of knockout mutants of endocytosis‐related genes. (A) Phenotypes of VdΔ*capa*, VdΔ*end3* and VdΔ*capaend3*. (B) Analysis of the penetration ability of VdΔ*capa*, VdΔ*end3*, and VdΔ*capaend3*. Images were taken before (above) and after (below) the cellulose membranes were removed. (C) Analysis of the pathogenicity of VdΔ*capa*, VdΔ*end3*, and VdΔ*capaend3*. Photographs were taken 20 days after infection.

Supporting information.

Supporting information.

## Data Availability

The data that support the findings of this study are available from the corresponding authors upon reasonable request.
