# Supplementary material for: The endocytic pathway for absorption of exogenous RNAs in Verticillium dahliae
Source: mLife. 2025 Feb 7;4(1):45–54. doi: 10.1002/mlf2.12149 (PMC11868834; doi:10.1002/mlf2.12149)
Supplement: Supplementary file 1 — Figure S1. Identification of V. dahliae knockout mutants of endocytosis‐related genes. (A) Protein structures of VdCapA and VdEND3. Pink represents the low‐complexity region, purple represents the EH domain, and green represents the coiled‐coil domain. (B) Southern blot analysis of knockout mutants. The restriction enzymes BamHI and EcoRI were used to digest genomic DNA. Figure S2. Phenotype and pathogenicity of knockout mutants of endocytosis‐related genes. (A) Phenotypes of VdΔcapa, VdΔend3 and VdΔcapaend3. (B) Analysis of the penetration ability of VdΔcapa, VdΔend3, and VdΔcapaend3. Images were taken before (above) and after (below) the cellulose membranes were removed. (C) Analysis of the pathogenicity of VdΔcapa, VdΔend3, and VdΔcapaend3. Photographs were taken 20 days after infection. [file MLF2-4-45-s003.pdf]

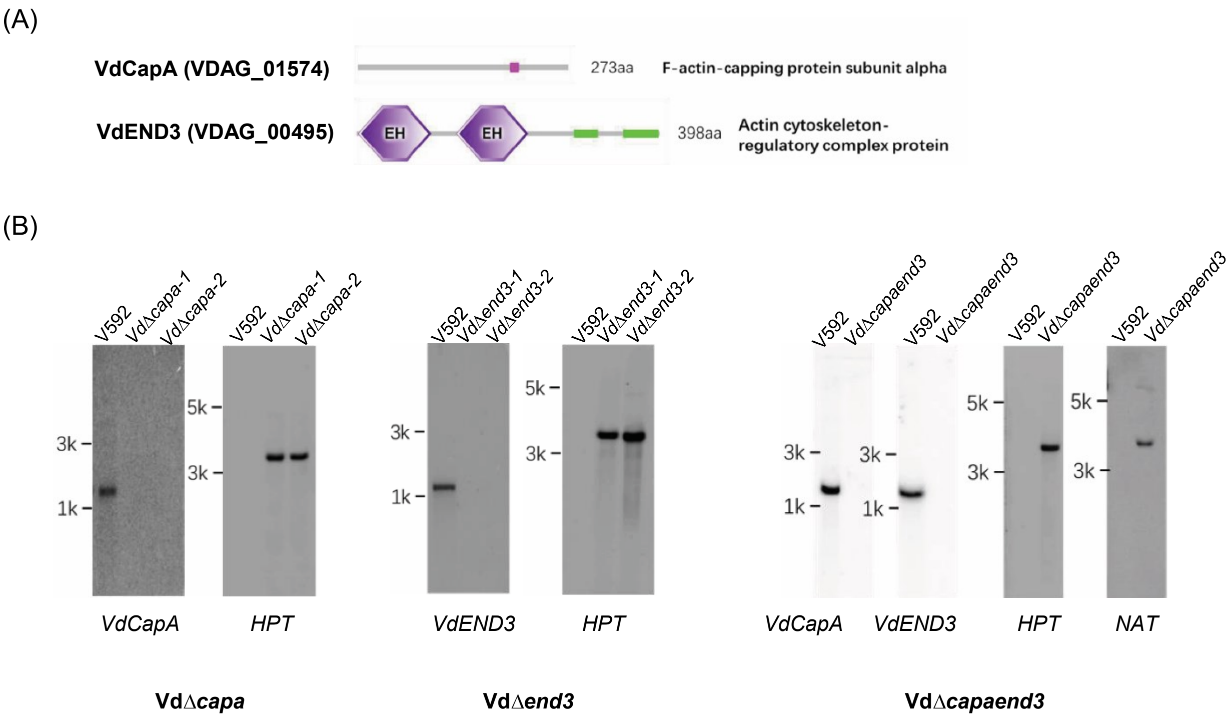
(A)
VdCapA (VDAG_01574)
273aa
F-actin-capping protein subunit alpha
VdEND3 (VDAG_00495)
EH
EH
398aa
Actin cytoskeleton-regulatory complex protein
(B)
V592
VdΔcapa-1
VdΔcapa-2
3k
1k
5k
3k
VdCapA
HPT
Vd∆capa
V592
VdΔend3-1
VdΔend3-2
3k
1k
5k
3k
VdEND3
HPT
Vd∆end3
V592
VdΔcapaend3
3k
1k
5k
3k
VdCapA
VdEND3
HPT
NAT
Vd∆capaend3

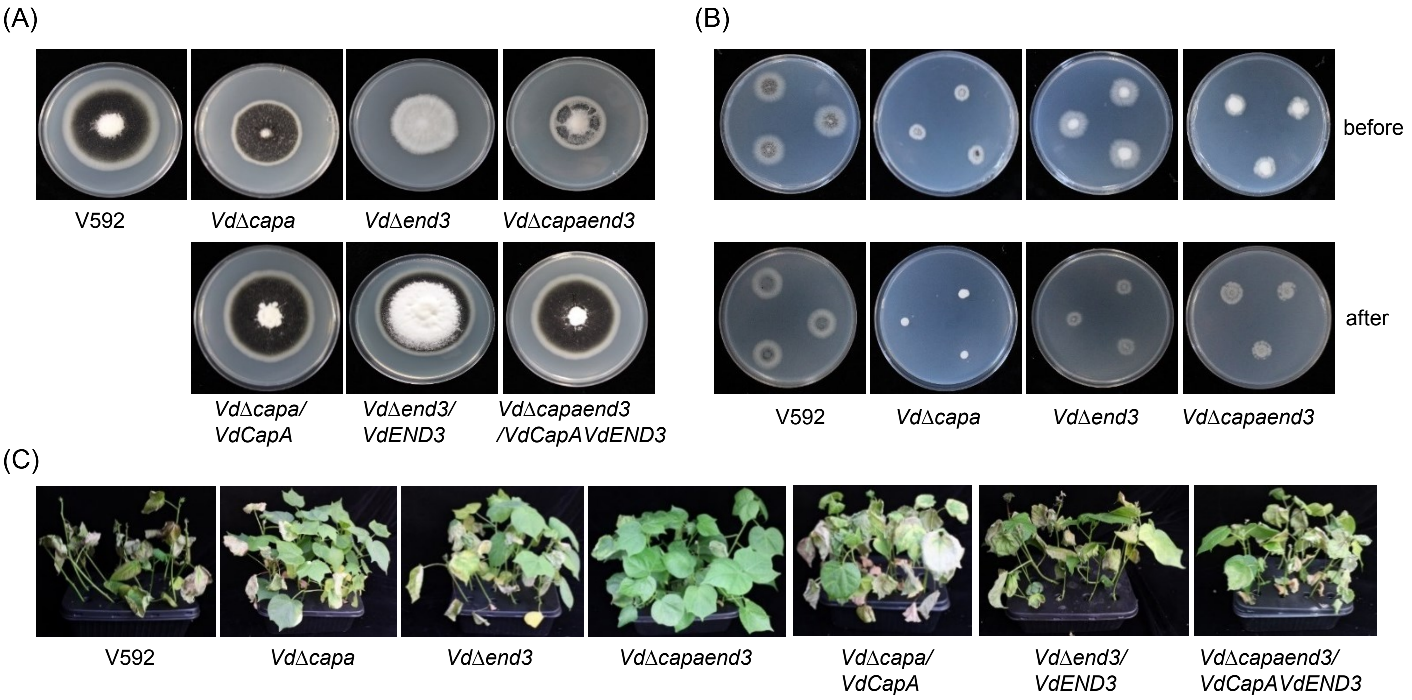

(A)
V592
VdΔcapa
VdΔend3
VdΔcapaend3
VdΔcapa/
VdCapA
VdΔend3/
VdEND3
VdΔcapaend3
/VdCapAVdEND3
(B)
before
after
V592
VdΔcapa
VdΔend3
VdΔcapaend3
(C)
V592
VdΔcapa
VdΔend3
VdΔcapaend3
VdΔcapa/
VdCapA
VdΔend3/
VdEND3
VdΔcapaend3/
VdCapAVdEND3
