# Supplementary material for: The endocytic pathway for absorption of exogenous RNAs in Verticillium dahliae
Source: mLife. 2025 Feb 7;4(1):45–54. doi: 10.1002/mlf2.12149 (PMC11868834; doi:10.1002/mlf2.12149)
Supplement: Supplementary file 2 — Supporting information. [file MLF2-4-45-s001.docx]

| **Primer names** | **Nucleotide Sequence (5’-3’)** | **Use in this study** |
| --- | --- | --- |
| VdH1-350-F | ACTCTCCAGACTTACATTGAT | T7-VdH1 |
| VdH1-350-R | CGATTTCGCTGATTCCCAGGG |  |
| RFP-717-F | ATGGTGTCTAAGGGCGAAGAG | T7-RFP |
| RFP-717-R | ATTAAGTTTGTGCCCCAGTTT |  |
| RFP-500-F | ATGGTGTCTAAGGGCGAAGAG | T7-RFP500 |
| RFP-500-R | TCAGGGCCATGTCGGTTCTGC |  |
| VdVPS-CDS-F | TCACAATCGATCCAATTAATTAACATGATGGAGGGATCCGGAAACT | pOlic-VdVPS-EGFP/ RFP |
| VdVPS-CDS-R | AGTGCCGCCTCCGCCGGATCCGCAGGCGCAACTGTCCTTGGC |  |
| VdYPTC5-CDS-F | TCACAATCGATCCAATTAATTAACATGTCGACGAGGAAGAAGGTC | pOlic-VdYPTC5-EGFP/ RFP |
| VdYPTC5-CDS-R | AGTGCCGCCTCCGCCGGATCCACAGGCACAGCCGTCGCGATC |  |
| VdCapA-up-F | GGGTTTAAUTGAAGGAGGGCAAGGACGTCA | pGKO-HPT-VdCapA |
| VdCapA-up-R | GGACTTAAUGGTGTATTGGGAGTTGTGCAC |  |
| VdCapA-down-F | GGCATTAAUGGTGGCCTGAGGCGAGCTAGG |  |
| VdCapA-down-R | GGTCTTAAUCTCTTGGTTTCATCGCGGGGAGC |  |
| VdEND3-up-F | GGGTTTAAUTATCACCAAAGCCCTTCCAGG | pGKO-HPT/Nat-VdEND3 |
| VdEND3-up-R | GGACTTAAUGTGTAGAGGTAGATCGGATAG |  |
| VdEND3-down-F | GGCATTAAUGGCCATGATTTGGATTCAACA |  |
| VdEND3-down-R | GGTCTTAAUCAACTTCTCGGCGGCGACTCA |  |
| VdCapA-CDS-Tef-F | CGTCAAACCTCTAGAGGATCCATGTCTGAGGTCGAGACTGTC | pTef-VdCapA-Neo |
| VdCapA-CDS-Tef-R | AGTGCCGCCTCCGCCGAATTCCCGCCGCGAGCCGCTGCCACC |  |
| VdCapA-CDS-Olic-F | TCACAATCGATCCAATTAATTAACATGTCTGAGGTCGAGACTGTC | pOlic-VdCapA-EGFP/RFP |
| VdCapA-CDS-Olic-R | AGTGCCGCCTCCGCCGGATCCCCGCCGCGAGCCGCTGCCACC |  |
| VdEND3-CDS-Tef-F | CGTCAAACCTCTAGAGGATCCATGGCCCCCCGAATCGAGCCG | pTef-VdEND3-Neo/Chl |
| VdEND3-CDS-Tef-R | AGTGCCGCCTCCGCCGAATTCTCGATTGACCTTTTCATTCTC |  |
| VdEND3-CDS-Olic-F | TCACAATCGATCCAATTAATTAACATGGCCCCCCGAATCGAGCCG | pOlic-VdEND3-EGFP/RFP |
| VdEND3-CDS-Olic-R | AGTGCCGCCTCCGCCGGATCCTCGATTGACCTTTTCATTCTC |  |
|  |  |  |
| VdCapA-F | ATGTCTGAGGTCGAGACTGTC | Southern blot |
| VdCapA-R | CCGCCGCGAGCCGCTGCCACC |  |
| VdEND3-F | ACGCAGCTCGAGCGAGTATGG | Southern blot |
| VdEND3-R | CGAGGGATTCGTAGAGGTCTT |  |
| HPT-F | TTCTGCTTCGCCGGAGCCTGA | Southern blot |
| HPT-R | GGTTGACGGCAATTTCGATGAT |  |
| Nat-F | GACAAGATGGTTCATTTAGGC | Southern blot |
| Nat-R | CACAGAGGCCGCAGAATGTGCT |  |
| miR166 | GGGGAATGAAGCCTGGTCCGA | Northern blot |
| VdClp1-F | ATGCACGGCTACAGCTCCTCCG | Northern blot |
| VdClp1-R | CTAGCAGAGCTCCTTCTCAGAC |  |

**Supplementary Table 1. List of primer and probe sequences used in this study**
