# Supplementary material for: The endocytic pathway for absorption of exogenous RNAs in Verticillium dahliae
Source: mLife. 2025 Feb 7;4(1):45–54. doi: 10.1002/mlf2.12149 (PMC11868834; doi:10.1002/mlf2.12149)
Supplement: Supplementary file 3 — Supporting information. [file MLF2-4-45-s002.docx]

**Figure S1.** Identification of *V. dahliae* knockout mutants of endocytosis-related genes. (A) Protein structures of VdCapA and VdEND3. Pink represents the low-complexity region, purple represents the EH domain, and green represents the coiled-coil domain. (B) Southern blot analysis of knockout mutants. The restriction enzymes *Bam*HI and *Eco*RI were used to digest genomic DNA.

**F****igure S2.** Phenotype and pathogenicity of knockout mutants of endocytosis-related genes. (A) Phenotypes of Vd*Δcapa*, Vd*Δend3* and Vd*Δcapaend3*. (B) Analysis of the penetration ability of Vd*Δcapa*, Vd*Δend3* and Vd*Δcapaend3*. Images were taken before (above) and after (below) the cellulose membranes were removed. (C) Analysis of the pathogenicity of Vd*Δcapa*, Vd*Δend3* and Vd*Δcapaend3*. Photographs were taken 20 days after infection.
